# Differences in the epigenetic and reprogramming properties of pluripotent and extra-embryonic stem cells implicate chromatin remodelling as an important early event in the developing mouse embryo

**DOI:** 10.1186/1756-8935-3-1

**Published:** 2010-01-12

**Authors:** Joana Santos, C Filipe Pereira, Aida Di-Gregorio, Thomas Spruce, Olivia Alder, Tristan Rodriguez, Véronique Azuara, Matthias Merkenschlager, Amanda G Fisher

**Affiliations:** 1 Lymphocyte Development Group, MRC Clinical Sciences Centre, Imperial College School of Medicine, Hammersmith Campus, Du Cane Road, London W12 0NN, UK; 2 Molecular Embryology Group, MRC Clinical Sciences Centre, Imperial College School of Medicine, Hammersmith Campus, Du Cane Road, London W12 0NN, UK; 3 Epigenetics and Development Group, Institute of Reproductive and Developmental Biology, Imperial College School of Medicine, Hammersmith Campus, Du Cane Road, London W12 0NN, UK

## Abstract

**Background:**

During early mouse development, two extra-embryonic lineages form alongside the future embryo: the trophectoderm (TE) and the primitive endoderm (PrE). Epigenetic changes known to take place during these early stages include changes in DNA methylation and modified histones, as well as dynamic changes in gene expression.

**Results:**

In order to understand the role and extent of chromatin-based changes for lineage commitment within the embryo, we examined the epigenetic profiles of mouse embryonic stem (ES), trophectoderm stem (TS) and extra-embryonic endoderm (XEN) stem cell lines that were derived from the inner cell mass (ICM), TE and PrE, respectively. As an initial indicator of the chromatin state, we assessed the replication timing of a cohort of genes in each cell type, based on data that expressed genes and acetylated chromatin domains, generally, replicate early in S-phase, whereas some silent genes, hypoacetylated or condensed chromatin tend to replicate later. We found that many lineage-specific genes replicate early in ES, TS and XEN cells, which was consistent with a broadly 'accessible' chromatin that was reported previously for multiple ES cell lines. Close inspection of these profiles revealed differences between ES, TS and XEN cells that were consistent with their differing lineage affiliations and developmental potential. A comparative analysis of modified histones at the promoters of individual genes showed that in TS and ES cells many lineage-specific regulator genes are co-marked with modifications associated with active (H4ac, H3K4me2, H3K9ac) and repressive (H3K27me3) chromatin. However, in XEN cells several of these genes were marked solely by repressive modifications (such as H3K27me3, H4K20me3). Consistent with TS and XEN having a restricted developmental potential, we show that these cells selectively reprogramme somatic cells to induce the *de novo *expression of genes associated with extraembryonic differentiation.

**Conclusions:**

These data provide evidence that the diversification of defined embryonic and extra-embryonic lineages is accompanied by chromatin remodelling at specific loci. Stem cell lines from the ICM, TE and PrE can each dominantly reprogramme somatic cells but reset gene expression differently, reflecting their separate lineage identities and increasingly restricted developmental potentials.

## Background

After fertilization, the mouse embryo undergoes a series of sequential cleavage divisions producing an eight-cell embryo, where blastomeres maximize their contact with one another in order to generate a compact sphere of cells. Subsequently, apico-basal polarization and asymmetric divisions generate two distinct cell populations at the 16-cell stage: large peripheral polarized cells and small apolar central cells [1]. The outer, polar cells of the late morula change morphology to form an epithelial monolayer of cells - the trophectoderm (TE), which mediates the implantation and initiation of placentation, while the inner apolar cells become the inner cell mass (ICM) and contain the founder cells of the embryo proper. By the early blastocyst stage (E3.5), these two tissues are morphologically distinct - the outer polarized epithelium, the TE, enclosing the ICM, which is itself heterogeneous [2]. Around the time of implantation, cells within the ICM segregate spatially and morphologically into the epiblast (EPI) and PrE lineages, through the migration of PrE cells to the blastocoelic surface of the ICM. Lineage studies have shown that the cells of the EPI are pluripotent and give rise to all tissues of the fetus plus extra-embryonic mesoderm. TE cells are multipotent differentiating exclusively into the trophoblast lineages that form the majority of the fetal placenta, while the PrE give rise to the visceral and parietal endoderm layers that will later line the yolk sack. Besides providing growth support and protection within the uterus, the extra-embryonic TE and PrE are sources of signals to the embryonic lineages to promote correct patterning and differentiation [3].

While the molecular mechanisms underlying the generation of the ICM, TE and PrE lineages are not fully understood, several transcription factors that play a role in the development of these three different lineages have been described, including Oct4, Cdx2 and Gata6, which are critical for the development of the ICM, TE and PrE, respectively [4-6]. An appropriate segregation of the ICM and TE has, in addition, been shown to be dependent upon the establishment and maintenance of cell polarity, involving E-cadherin and the Par3/aPKC complex [7-9].

Studies from several laboratories have provided evidence of global epigenetic differences between these early lineages that may be important in defining their developmental fate. In particular, a recent study has suggested that at the four-cell-stage mouse embryo, blastomeres with higher levels of histone H3 arginine methylation are more likely to contribute to the pluripotent cells of the ICM [10]. Moreover, while the TE (and also the PrE) are hypomethylated both at repetitive and structural gene sequences [11,12] throughout development, a striking increase in both DNA and H3K9 methylation levels characterizes the ICM at the blastocyst stage [13,14]. In addition, epigenetic asymmetry between embryonic and extra-embryonic tissues is evident during X-inactivation, which is random in embryonic but imprinted in the TE and PrE lineages [15,16].

**Figure 1:**
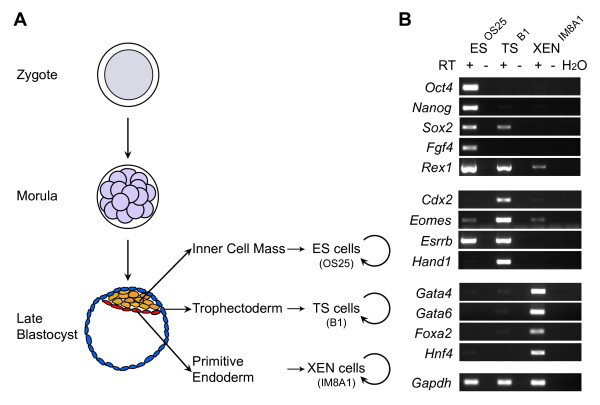
**Embryo-derived stem cell lines respectively express lineage-associated markers**. (A) Brief schematic representation of mouse pre-implantation development and origin of lineage-derived stem cells. Embryonic stem (ES), trophectoderm stem (TS) and extra-embryonic endoderm (XEN) can be derived from the inner cell mass (yellow), trophectoderm (blue) and primitive endoderm (red) of mouse blastocysts. (B) Reverse transcription polymerase chain reaction expression analysis of ES^OS25^, TS^B1 ^and XEN^IM8A1 ^cells lines. RNA was isolated and cDNA prepared from all three embryo-derived stem cell lines cells and analysed using primers for *Oct4*, *Nanog*, *Sox2*, *Fgf4*, *Rex1*, *Cdx2*, *Eomes*, *Esrrb*, *Hand1*, *Gata4*, *Gata6*, *Foxa2*, *Hnf4 *and *Gapdh *as a loading control. +/- indicates presence or absence of reverse transcriptase; H_2_O, water control.

Recently, data from two different sources has provided important insights into how lineage potential is regulated at the earliest stages of mammalian development. Studies comparing DNA methylation at gene promoters in embryonic stem (ES) versus trophectoderm stem (TS) cells, germ cells and fibroblasts identified novel factors that act as 'gatekeepers' for the specification of extra-embryonic tissue [17] and showed that epigenetic reprogramming, essential for the transmission of pluripotency, occurs within the germline prior to fertilization [18]. Another set of reports, in which the chromatin profile of ES cells, somatic stem cells and their differentiated progeny were contrasted, provided collective evidence that many developmental regulators genes in ES cells are primed for future expression, being marked with histone modifications associated with both active and repressed chromatin [19,20]. In this study we have examined the epigenetic status of other blastocyst-derived lineages required for the successful development of the early mammalian embryo, using stem cells lines isolated from the TE [21] and PrE [22] that self-renew and differentiate into defined extra embryonic tissues (Figure 1A). Our results demonstrate that, following lineage specification to the ICM, TE and PrE, there are predictable changes in the temporal replication and chromatin structure of lineage-determining genes, as reflected in the stem cell lines analysed here. We also show that extra-embryonic endoderm (XEN) and TS cells, like ES cells, can dominantly reprogramme somatic cells (human lymphocytes), but that they initiate discrete and different lineage-specific gene expression programmes. Taken together, these results suggest that dynamic changes in chromatin organization occur within the developing blastocyst and that these epigenetic changes are important for cell specification and conveying lineage identity.

## Results

### ES (OS25), TS (B1) and XEN (IM8A1) cells selectively express genes characteristic of either the ICM, trophectoderm or primitive endoderm

Reverse transcription polymerase chain reaction (RT-PCR) was used to assess the relative abundance of different mRNA transcripts in ES (OS25, [23]), TS (B1, [21]) and XEN (IM8A1, [22]) cell lines. Consistent with previous reports, ES cells expressed *Oct4*, *Nanog*, *Sox2*, *Fgf4*, *Rex1 *[24] and *Esrrb *[25], TS cells expressed *Cdx2*, *Eomes*, *Esrrb *and *Hand1 *[21], and XEN cells selectively expressed *Gata4*, *Gata6*, *Foxa2 *and *Hnf4 *[22] (Figure 1B and Additional file 1). *Rex1 *transcripts were detected in all three cell types but were most abundant in ES cells; *Sox2 *transcripts were detected in both ES and TS cells; *Eomes *transcripts were detected in all three cell types but were most abundant in TS cells. These data show that each of the stem cell lines displays a different profile of gene expression, in line with previous studies [21,22,24] and with their different origins. At the level of specific genes, however, there is considerable overlap in expression between the cell lines.

### ES, TS and XEN cell lines have similar but distinct replication timing profiles

**Figure 2:**
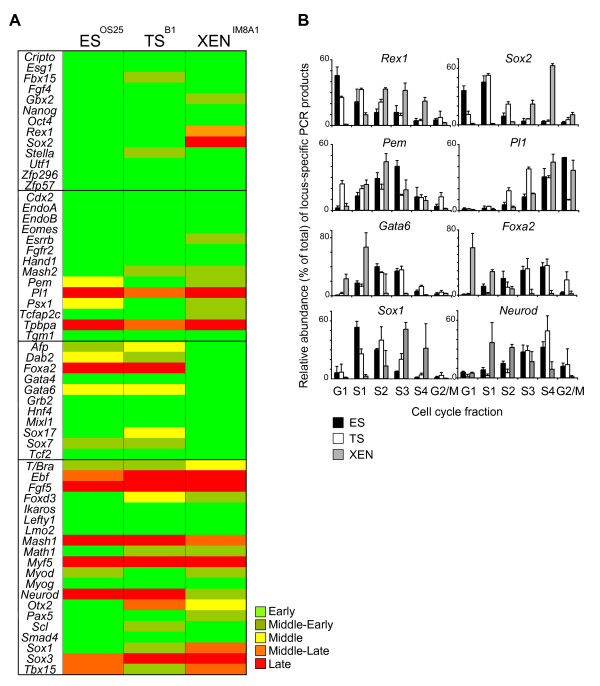
**Embryonic stem (ES), trophectoderm (TS) and extra-embryonic endoderm (XEN) cell populations have distinct replication timing profiles, which reflect their lineage potential**. (A) Summary of the replication timing comparison of the selected candidate genes between the three embryo-derived stem cell lines. The replication timing of each gene was defined according to its peak abundance in G1/S1 (early, dark green), S2 (middle-early, light green), S2 and S3 (middle, yellow), S3 (middle-late, orange) or S4/G2 (late, red), determined in at least two independent experiments. Inner cell mass/ES-, TE/TS-, PrE/XEN-related *loci *or genes involved in the specification of somatic cell types are grouped into four different boxes. (B) Histograms comparing the relative abundance of *locus*-specific signal for *Rex1*, *Sox2*, *Pem*, *Pl1*, *Gata6*, *Foxa2*, *Sox1 *and *Neurod loci *within each cell cycle fraction for ES (black bars), TS (white bars) and XEN (grey bars) cells as assessed by quantitative polymerase chain reaction. Mean and standard deviation of two or more experiments are shown for each cell type analysed.

In order to directly compare the epigenetic profiles of extra-embryonic stem cell lines with those of pluripotent cell lines, we initially assessed the replication timing of a panel of developmental genes in OS25, B1 and IM8A1 cell lines. Genes include those that encode transcription factors regulating the specification of germ layers in the embryo [19], as well as those encoding transcription factors that are important for the biology of early embryonic ICM, TE, PrE and EPI lineages. Replication was assessed using a previously established assay [26,27] in which asynchronous cells are pulse-labelled with 5-bromo-2-deoxyuridine (BrdU), fractionated according to cell-cycle stage (see Additional file 2, part A) and the relative abundance of newly synthesized *locus*-specific DNA is compared between successive cell cycle fractions using quantitative PCR. Although the exact relationship between chromatin structure and replication timing is not fully understood, early replication is a characteristic of 'accessible' and highly acetylated chromatin while late replication is a feature of heterochromatic domains and some repressed genes [28]. Consistent with this, α-*globin *a constitutively early replicating gene, was detected in S1 fractions isolated from ES, TS and XEN cells (Additional file 2, part B top panel), while *Amylase 2.1*, a late replicating control, was detected in S3 and peaked in the S4 fractions in all three cell types (Additional file 2, part B middle panel) [19]. Detection of similar levels of BrdU-labelled *Gbe *DNA in cell cycle fractions that were 'spiked' with a constant amount of *Drosophila *BrdU-labelled DNA (Additional file 2, part B lower panel), confirmed an equivalent recovery of immuno-precipitated DNA in all analyses shown. The replication times of candidate genes were determined from at least two independent experiments, scored according to a peak abundance of *locus*-specific DNA (in G1/S1 [early], S2 [middle-early], S2 and S3[middle], S3 [middle-late] or S4/G2 [late]) and the results were colour-coded to facilitate comparison (see Figure 2, as previously described [19,27]).

Most of the genes analysed replicated in early (or middle-early) S-phase in all three stem cell lines (44, 44, 45 out of 58 genes in ES, TS and XEN cells, respectively, Figure 2A). These included a subset of ICM-associated genes (*Oct4*, *Nanog *and *Fgf4*) expressed by ES cells, as well as genes associated with TE (*Cdx2 *and *Hand1*) and PrE (*Gata4 *and *Hnf4*). In addition, many genes that are not thought to be expressed at significant levels in any of these cell types, for example *Math1*, *Scl *and *Myog*, replicate early in all three embryonic stem cell lines. These data suggests that in TS and XEN cells, many developmental regulator genes remain 'accessible' - as reflected by the prevalence of early replicating *loci *- similar to that reported previously for ES cells [19]. Overall, the replication timing profiles of ES and TS cells were similar (41/58) or identical (32/58), while XEN cells showed a greater disparity. This is illustrated by a delayed replication of several pluripotency-associated genes in XEN cells (for example, *Rex1 *and *Sox2) *and the early replication of PrE-associated genes *Gata6 *and *Foxa2 *(Figure 2B) and is in keeping with the idea that some tissue-specific genes may replicate earlier when transcriptionally active [29,30]. Similarly, *Pem *and *Psx1*, which encode factors required for extra-embryonic lineages, replicated later in ES cells as compared to TS and XEN cells and the replication of *Pl1*, a TE-specific factor, was selectively advanced in TS cells (Figure 2B). These results were confirmed by analysing additional independent TS and XEN cell lines (Additional file 1) that were derived from mice carrying floxed Dicer alleles [31]. Comparing TS^B1 ^and TS^Dicerfx/fx ^or XEN^IM8A1 ^and XEN^Dicerfx/fx ^(Figure 2 and Additional file 1), as well as numerous different ES cell lines [19,32], confirmed that the replication timing profiles of different embryonic and extra-embryonic cell lines were robustly preserved.

Interestingly, the neural-associated genes *Sox1 *and *Neurod *that are not expressed by any of the embryonic stem cell lines, showed clear differences in replication timing between ES, TS and XEN cells (Figure 2B, lower panel). *Sox1 *replication was advanced in ES cells while *Neurod *replicated early in XEN cells. Although unexpected, these results suggest underlying changes in the chromatin context of these genes in the stem cell lines. In the case of *Neurod*, although the transcription factor is known to function in neuronal development, it has also been shown to have an important role in the development of specialized cell types arising from the gut endoderm [33]. Despite being derived from the EPI and not from the PrE, gut endoderm cells have morphological and functional similarities to visceral endoderm cells [34]. The advanced replication of *Neurod *in XEN cells might therefore reflect changes in transcriptional competence at the *locus *that is associated with an affiliation to the 'endoderm' lineage.

### Chromatin profiling of gene promoters in stem cell lines

The chromatin profile of important regulator genes was compared between embryo-derived stem cell lines using chromatin immunoprecipitation (ChIP) in order to evaluate the abundance of specific histone modifications that are associated with either active (H3K4me2, H4ac and H3K9ac) or repressed (H3K27me3 and H4K20me3) chromatin. For these analyses primers were designed to recognize the promoter region (up to 600 kb upstream the transcriptional start site) of each candidate gene; genes that are known to be abundantly expressed by each cell type were used as positive controls for 'active' chromatin marks. Pericentric heterochromatin (γ-satellite repeats) provided controls for H4K20me3 immuno-precipitations, H3K27me3 was validated by analysing known bivalent *loci *in ES cells [19], and the abundance of modified histones was calculated relative to histone H3.

**Figure 3:**
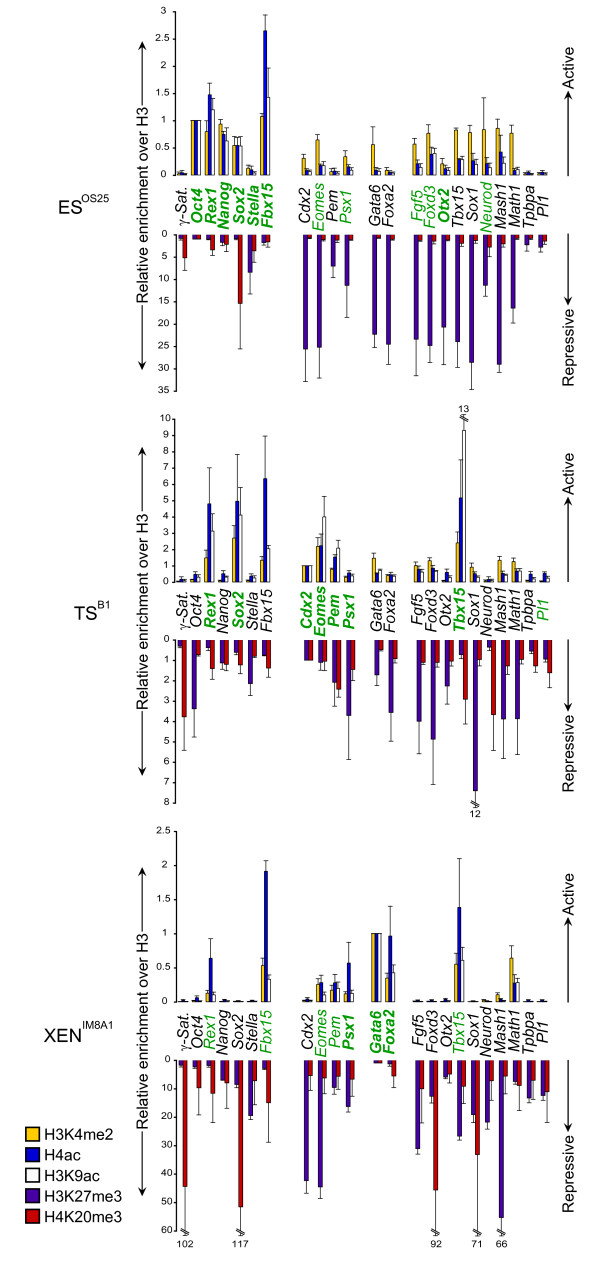
**Histone modifications at the promoters of key developmental regulator genes in embryonic stem (ES), trophectoderm stem (TS) and extra-embryonic endoderm (XEN) cells**. The abundance of active [histone 3 lysine 4 dimethylation (H3K4me2, yellow bars), histone 4 acetylation (H4ac, blue bars), histone 3 lysine 9 acetylation (H3K9ac, white bars)] and repressive [histone 3 lysine 27 trimethylation (H3K27me3, purple bars), histone 4 lysine 20 trimethylation (H4K20me3, red bars)] histone marks at selected *loci *was assessed in ES, TS and XEN cells by chromatin immunoprecipitation and quantitative polymerase chain reaction. Values are shown as the ratio of modified histone H3 to unmodified histone H3 immunoprecipitations and normalized to an abundantly expressed gene in each cell type; *Oct4 *in ES cells, *Cdx2 *in TS cells and *Gata6 *in XEN cells. Detected transcripts are highlighted in green while overt gene expression is shown in bold green. Primers were designed to the promoter region (100-600 bp upstream the transcriptional start site). Error bars represent the standard deviation of three independent experiments.

As anticipated, the promoters of many genes that are overtly expressed in ES cells (shown in bold, Figure 3, upper panel), as well as many bivalent genes (including *Eomes*, *Fgf5*, *Foxd3*, *Mash1*, *Math1*, *Sox1 *and *Tbx15) *were enriched for H3K4me2, and/or H3K9ac and H4ac at their promoters [19,20]. Exceptions included the promoters of *Tpbpa *and *Pl1*, two markers of differentiated trophoblast. In ES cells histone H3K27me3, a modification catalyzed by polycomb repressor complex 2 (PRC2), was abundant at the promoters of genes that were either not expressed or expressed at low levels, including TS-associated genes (*Cdx2*, *Eomes*, *Pem*, *Psx1*), PrE-associated genes (*Gata6, Foxa2*) and genes that are normally expressed by subsets of differentiated tissue (such as *Mash1*, *Math1 *and *Neurod*) (shown in purple in Figure 3). Some silent late-replicating genes showed only low levels of H3K27 trimethylation (*Tpbpa *and *Pl1) *suggesting that these genes, in contrast to bivalent genes, are not developmentally 'poised' in ES cells and may, therefore, require extensive chromatin-remodelling for correct developmental expression. Levels of promoter H4K20me3 (shown in red in Figure 3), a mark associated with mammalian pericentric heterochromatin [35], were modest in ES cells with the exception of *Sox2 *(an observation that is likely to reflect the fact that OS25 cells carrying *Sox2 *as a transgene). In TS (B1) and XEN (IM8A1) cell lines, in contrast to ES (OS25), H4K20me3 was detected at the promoters of many genes and was particularly enriched at several silent genes in XEN cells (*Sox2, Foxd3, Sox1*) (Figure 3, see Additional file 3 and Figure 1A for expression data). Taken as a whole these ChIP analyses suggest that, although the promoters of many development regulator genes are co-marked with histone modifications associated with active (acetylated, H3K4me2) and repressive (H3K27me3) chromatin in both ES and TS cells, this is not the case in XEN cells. Rather, in XEN cells histone marks that characterize accessible chromatin genes tend to be restricted to genes that are productively expressed at high *(Gata6*, *Foxa2*, *Pem*, *Psx1*) or moderate levels (*Eomes*, *Fbx15*, *Rex1, Tbx15)*. The exception to this generalization is *Math1 *(lower panel of Figure 3 and Additional file 3), a promoter that is enriched for H3K4me2 in ES, TS and XEN cells and, therefore, appears to retain a bivalent (or poised) configuration. Although we do not currently know the cause or significance of this single observation, collectively our data suggest that the chromatin structure of many genes is different between the stem cell lines, supporting earlier proposals that epigenetic reprogramming occurs in embryonic and extra-embryonic lineages during early mouse development [17,18].

### Mouse TS, XEN and ES cell lines dominantly reprogram lymphocytes in interspecies heterokaryons but induce the expression of different lineage-associated genes

**Figure 4:**
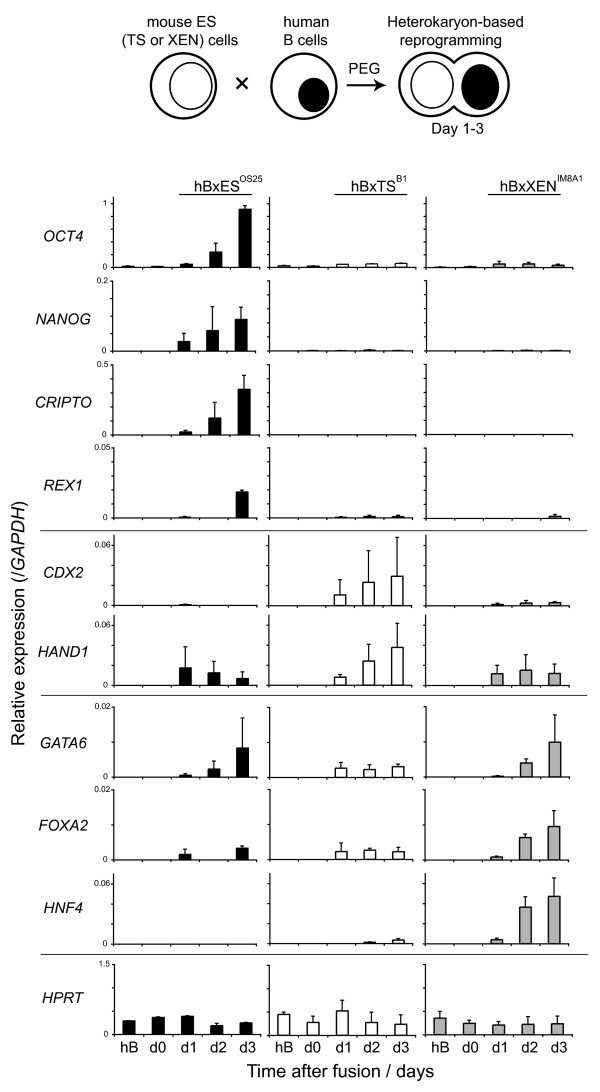
**Lineage restricted dominant reprogramming of human lymphocytes by embryo-derived stem cells**. The reprogramming activity of embryonic stem (ES), trophectoderm stem (TS) and extra-embryonic endoderm (XEN) cells was assessed by heterokaryon formation with human B-lymphocytes (hB). Relative expression levels of human stem cell-specific genes in interspecies heterokaryons were detected by quantitative reverse transcriptase polymerase chain reaction analysis. The transcript levels of ES (*OCT4*, *NANOG*, *CRIPTO*, *REX1*), TS (*CDX2*, *HAND1*) and XEN-specific (*GATA6*, *FOXA2 *and *HNF4) *genes were measured 1 to 3 days after fusion and normalized to *GAPDH *expression. The constitutively expressed gene *HPRT *was included as a control. Values shown are the mean from two independent experiments and error bars indicate standard deviations.

Spontaneous and experimental cell fusions between ES cells and cells from a range of somatic tissues result in the nuclei of differentiated cells being reprogrammed to express an ES-specific gene expression pattern (so-called dominant reprogramming) [36,37]. Although the rules of dominance are not fully understood, other stem cell populations, including embryonic germ and embryonic carcinoma cells, can also reprogramme somatic cells towards a pluripotent state *in vitro *[38,39]. In order to establish whether extra-embryonic stem cell populations have this capacity and, moreover, whether reprogramming by these extra-embryonic cells induces the *de novo *expression of different cohorts of genes, we tested the ability of TS and XEN cells in order to reprogramme human B cells using a previously established assay system [40]. Briefly, mouse stem cells and human B-lymphocytes were mixed in a 1:1 ratio and inter-species heterokaryons (cells in which parental nuclei share the same cytoplasm but remain spatially separate) were generated by polyethylene glycol (PEG)-mediated cell fusion. Human B cell reprogramming within these heterokaryons was assessed 1 to 3 days after fusion, by qRT-PCR using primers that were designed (and validated) to specifically amplify human transcripts. Expression of *HPRT *served as a positive control in these analyses and transcripts derived from the human pluripotency-associated genes (*OCT4*, *NANOG*, *CRIPTO *and *REX1*), TE (*CDX2 *and *HAND1*) and PrE-associated genes were examined in detail (*GATA6*, *FOXA2 *and *HNF4*) (Figure 4). Human B cells (hB) did not express detectable levels of pluripotency-associated transcripts prior to fusion, but following heterokaryon formation with mouse ES cells (hBxES), expression of *OCT4*, *NANOG*, *REX1 *and *CRIPTO *was initiated and increased up to day 3 (Figure 4, upper panel left). Fusion of hB with mouse TS (B1) or with mouse XEN (IM8A1) cells did not induce the expression of any of the human pluripotency-associated genes tested, including *CRIPTO*. This could be considered surprising as XEN (IM8A1) cells express high levels of mouse *Cripto *transcripts (several fold more than ES cells) and both TS and XEN cell lines express mouse *Rex1 *(Additional file 4). In heterokaryons formed between hB and TS (B1), expression of TE-associated genes (*CDX2 *and *HAND1) *was induced, as well as low levels of some PrE-associated genes (*GATA6 *and *FOXA2*). Fusions between hBxXEN cells resulted in a rapid and sustained induction of *GATA6*, *FOXA2 *and *HNF4 *(Figure 4). In contrast, mouse lymphocyte-specific transcripts (such as *CD19*, *CD37 *and *CD45*) were not detected throughout these experiments (data not shown) which is in line with the dominance of embryonic and extra-embryonic stem cells in reprogramming. These results collectively show that XEN and TS stem cell lines, like ES cells, retain a capacity to dominantly reprogramme somatic cells, but impose a programme of gene expression that is consistent with their different lineage affiliations.

## Discussion

In this study we show that stem cell lines derived from the ICM, TE and PrE, display distinct epigenetic properties as defined by replication timing, chromatin profiling and reprogramming potential. However, our studies revealed that many genes that are important in determining cellular fate are retained in an 'accessible' chromatin state (acetylated and early replicating) in trophoblast-restricted stem cells (TS), being co-marked also by PRC2-mediated H3K27me3. This chromatin configuration, often referred to as 'bivalent', is shared with ES cells [19,20] and results in non-productive gene expression [41,42]. It is thought to be important for priming specific cohorts of genes for future developmental expression [19,43,44], and may therefore be important for restraining differentiation [41,45,46]. In keeping with this idea, our data show that few developmental regulator genes appear to be primed (bivalent) in XEN cells as compared with ES or TS cells, perhaps reflecting their narrower developmental potential. In addition, the delayed replication of several neuronal-associated genes in XEN cells relative to ES (for example *Otx2*, *Sox1 *and *Sox2*), infers a change in chromatin status and the loss of promoter acetylation. Similar delays have been reported in mature B and T-lymphocytes [19], which, like XEN cells, have a more restricted (non-neuronal) fate and also in the case of F9 embryonic carcinoma cells, which show a propensity to differentiate to endoderm lineages [47]. Interestingly, *Sox1, Sox2 *and the neural crest marker *Foxd3 *also display high levels of H4K20me3 levels at their promoters in XEN cells, consistent with reduced transcriptional competence. The exception to this general trend is *Math1*, a gene that appears to be functionally primed in XEN cells being simultaneously enriched for 'active' histone marks (H3K4me2, H3K9ac and H4ac) as well as 'repressive' PRC2-mediated H3K27me3. As this gene is known to be involved in the generation of the secretory cell lineages in the intestine [48] that are derived from the definitive endoderm, it is conceivable that *Math1 *has a conserved role in the development of extra-embryonic endoderm lineages. Consistent with this idea, XEN cells exhibit a strong bias to form parietal endoderm in chimeras, a tissue which is highly specialized for the synthesis and secretion of extracellular matrix proteins [22,49].

The similar histone modifications and replication timing profiles between ES and TS cells is consistent with mounting evidence indicating that only relatively few genes are uniquely restricted to the placenta, the vast majority of candidate TS-associated genes being involved in the development of other organs within the embryo proper [50]. Some genes such as *Oct4 *and *Nanog*, which are downregulated in TS cells (but remain early-replicating), probably rely on alternative epigenetic mechanisms to suppress transcription in extra-embryonic lineages. For example, in TS cells *Oct4 *and *Nanog *regulatory domains are hyper (DNA) methylated and hypoacetylated, relative to ES cells [51,52]. Despite the overall similarity between ES and TS, the use of a candidate-based replication timing assay allows *loci *that are subject to chromatin re-modelling events early in mammalian development to be readily identified. A number of studies have suggested that the generation of the ICM and TE requires the development of cell polarity in the outer cells of the morula, and the linked asymmetric divisions of blastomeres at the eight-cell-stage [53]. The significance of this polarization event is reflected by the identification of *loci *involved in cell polarity and cytoskeleton dynamics among candidates that replicate earlier in TS cells than in ES cells (such as *Epb4.1l3*, *Fez2 *and *Cdh5*, data not shown) in addition to *Dab2*, which are likely to be functional relevant for the biology of the trophoblast lineage.

The reprogramming properties of extra-embryonic stem cells have, to our knowledge, received little attention. Here, experimental heterokaryons were generated to ask whether TS and XEN cells were capable of dominant reprogramming human somatic cells and, if so, whether they could impose different lineage-specific gene expression programmes. We demonstrate that TS and XEN cells reprogramme human B-lymphocytes in order to establish TE- or PrE-specific gene expression, respectively, albeit at low levels. As these fusions were performed using cells from different mammalian species, low expression levels may reflect inter-species differences, such as mismatches between mouse factors and *cis *acting elements within human genes [54]. Despite this, fusions using ES, TS or XEN cells reprogrammed human lymphocytes differently, the outcome reflecting discrete lineage affiliations. Interestingly, the expression of human transcripts by reprogrammed B-cell nuclei was not identical to that produced by the mouse stem cell eliciting the dominant reprogramming. This observation mirrors previous reports that fusion with mouse ES cells, results in human B cells expressing a human ES-specific gene expression profile (hSSEA4, hFGF2 and hFGFR1), while hallmark factors of mouse ES cells, such as Lif receptor and Bmp4, are not activated [38]. In this context, it seems likely that reprogrammed hB cells display features of human extra-embryonic-specific gene expression upon heterokaryon formation with mTS or mXEN cells, in agreement with published data [55]. Since extra-embryonic derived human stem cell lines have not been fully characterized, the generation of heterokaryon and hybrid cells using this approach could provide an important tool for studying human extra-embryonic lineages.

## Conclusions

This report provides a preliminary epigenetic characterization of mouse TE and PrE extra-embryonic lineages using stem cell lines as a model. We provide evidence of qualitative differences in the chromatin profiles between embryo-derived stem cell lines that accurately reflect their different transcriptional, lineage commitment and developmental potentials. These data support previous *in vivo *studies of pre-implantation stage embryos [13,14], showing that dynamic changes in chromatin occur at the earliest stages of mammalian development and are likely to be important for refining cellular potential.

## Methods

### Cell lines and cell culture

ES cells (OS25) were maintained in an undifferentiated state on 0.1% gelatin (StemCell Technologies, Vancouver, Canada)-coated flasks (Fisher Scientific UK Ltd, Leicestershire, UK) in G-MEM-BHK 21 medium (Invitrogen Ltd, Paisley, UK) supplemented with 10% fetal calf serum (FCS; PAA Laboratories, Gmbh, Pasching, Austria), non-essential amino acids, sodium pyruvate, sodium bicarbonate, antibiotics, L-glutamine, β-mercaptoethanol (Sigma-Aldrich Co Ltd, Gillingham, UK) and ESGRO-LIF (1000 U/ml) (Chemicon/Millipore, Billerica, USA). TS cell lines (B1 and Dicerfx/fx) were cultured in the presence of 70% mitotically inactivated mouse embryo fibroblast cells-conditioned medium and 30% TS medium to which human recombinant Fgf4 (25 ng/ml) (Sigma-Aldrich) and heparin (1 μg/ml) (Sigma-Aldrich) were added. The TS cell medium was RPMI 1640 supplemented with 20% FCS (GlobePharm, Cork, Ireland), sodium pyruvate, β-mercaptoethanol, L-glutamine and antibiotics. XEN cell lines (IM8A1 and Dicerfx/fx) were maintained on 0.1% gelatin-coated flasks in RPMI 1640 supplemented with 20% FCS (GlobePharm), sodium pyruvate, L-glutamine, antibiotics and β-mercaptoethanol. EBV-transformed human B-lymphocyte clones were maintained in RPMI medium supplemented with 10% FCS (GlobePharm), L-glutamine and antibiotics. All cell lines used in this study were subjected to karyotypic analysis to check chromosome number. XEN cell lines routinely contained 40-46 chromosomes consistent with their previously reported aneuploid status [22] while ES and TS cell lines appeared normal.

### RT-PCR analysis

RNA extraction from ES, TS, XEN cells and heterokaryons was performed using RNeasy protect mini kit (Qiagen, USA) and RNase-free DNase set (Qiagen) for digestion of residual DNA. Total RNA (2.5 μg) was then reverse transcribed using the Superscript first-strand synthesis system (Invitrogen) and cDNA of interest amplified in a total reaction volume of 50 μL using 500 nM primers, and 1.25 U of HotStarTaq (Qiagen). The PCR cycling conditions were as follows: 95°C for 2 min, 30 cycles 95°C for 30 s, annealing at 60°C or 65°C for 30 s and elongation at 72°C for 2 min, finishing with a step at 72°C for 10 min.

### Replication timing assay

BrdU-labelling, ethanol fixation, cell cycle fractionation by flow cytometry and isolation of BrdU-labelled DNA by immunoprecipitation were carried out as previously described [19] with the same BrdU-pulse labelling time for all three stem cell populations (30 min). The abundance of newly replicated DNA in each cell-cycle fraction was determined by real-time PCR amplification.

### Real-Time PCR analysis

Real-Time PCR analysis was carried out on a Opticon™ DNA engine (MJ Research, Inc, MA, USA) under the following cycling conditions: 95°C for 15 min, 40 cycles at 94°C for 15 s, 60°C for 30 s, 72°C for 30 s followed by plate read. PCR reactions were performed in a 30 μL reaction volume containing 2× SYBR Green (Qiagen), 1.5 μL of template and 300 nM primers. Each measurement was performed in duplicate. For heterokaryon analysis data were normalised to human *GAPDH *expression.

### Chromatin immunoprecipitation analysis

Exponentially growing ES, TS and XEN cells were processed for ChIP analysis as described previously [19]. 140 μg chromatin was subjected to immunoprecipitation with 5 μL anti-H3K9ac (Upstate Biotechnology, NY, USA), 5 μL anti-H3K4me2 (Upstate), 5 μL anti-H4ac (Upstate), 5 μL anti-H3K27me3 (Upstate), 5 μL anti-H4K20me3 (Upstate), 2.5 μL of a rabbit anti-mouse-IgG antiserum (negative-control) (Dako Inc, CA, USA) and 4 μL of anti-H3 (Abcam, MA, USA). After purification, DNA was resuspended in 80 μL TE solution. Quantification of precipitated DNA was performed using real-time qPCR (quantitative PRC) amplification. Histone's modification levels were normalized against total H3 detected and the ratio of modified-H3 to H3 was denoted as relative enrichment. ChIP experiments were performed twice.

### Experimental heterokaryons

Heterokaryons were generated by fusing either mouse ES, TS or XEN cells and human B-lymphocytes using 50% polyethylene glycol, pH 7.4 (PEG 1500, Roche, Hertfordshire, UK). Equal numbers of stem cells and B-lymphocytes were mixed, washed twice in phosphate buffered saline at 37°C and 1 mL of PEG at 37°C was added to the pellet of cells over 60 s followed by an incubation at 37°C for 90 s. Cell mixtures were washed with 10 mL of DMEM at 37°C added over 3 min. After centrifugation the pellet was allowed to swell in complete medium for 3 min before resuspension. In order to eliminate non-fused hB cells Ouabain (10^-5 ^M) was added to the medium. Proliferating stem cells were eliminated by the addition of 10^-5 ^M Ara-C 6 h after fusion and then removed after 12 h. Fused cells were cultured under conditions promoting the maintenance of undifferentiated mouse stem cells.

## Abbreviations

ChIP: chromatin immunoprecipitation; EPI: epiblast; ES: embryonic stem; FCS: fetal calf serum; hB: human B cells; ICM: inner cell mass; PBS: phosphate buffered saline; PCR: polymerase chain reaction; PEG: polyethylene glycol; PRC2: polycomb repressor complex 2; PrE: primitive endoderm; qPCR: quantitative PCR; RT: reverse transcription; TE: trophectoderm; TS: trophectoderm stem; XEN: extra-embryonic endoderm.

## Competing interests

The authors declare that they have no competing interests.

## Authors' contributions

JS and CFP contributed equally to this work. JS carried out the experiments with help from OA and analysed the data. CFP carried out the reprogramming and revision experiments with help from AD, TS and TR. VA, MM and AGF participated on the design of the study. All authors have read and approved the manuscript.

## Supplementary Material

Additional file 1 **Replication timing and gene expression analysis of additional trophectoderm stem (TS) and extra-embryonic endoderm (XEN) cell lines**. (A) Summary of the replication timing comparison of the selected candidate genes in TS^Dicerfx/fx ^and XEN^Dicerfx/fx ^stem cell lines. The replication timing of each gene was defined according to its peak abundance in G1/S1 (early, dark green), S2 (middle-early, light green), S2 and S3 (middle, yellow), S3 (middle-late, orange) or S4/G2 (late, red), determined in at least two independent experiments. Inner cell mass/ES-, trophectoderm/TS-, primitive endoderm/XEN-related *loci *or genes involved in the specification of somatic cell types are grouped into four different boxes. Comparison with data presented in Figure 2A shows that 50/58 and 52/58 showed identical replication times in TS and XEN cells, respectively. Of the remaining genes, 8/8 and 6/6 showed similar replication times (peak abundance in an adjacent cell cycle fraction) in these cell lines. (B) Reverse transcriptase polymerase chain reaction expression analysis of TS^Dicerfx/fx ^and XEN^Dicerfx/fx ^cell lines. RNA was isolated and cDNA analysed using primers for *Oct4*, *Nanog*, *Sox2*, *Fgf4*, *Rex1*, *Cdx2*, *Eomes*, *Esrrb*, *Hand1*, *Gata4*, *Gata6*, *Foxa2*, *Hnf4 *and *Gapdh *as a loading control. +/- indicates presence or absence of reverse transcriptase; H_2_O, water control. Click here for file

Additional file 2 **Replication timing analysis of embryo-derived stem cell lines**. (A) Typical cell cycle profiles for each cell population based on propidium iodide (PI) staining. Embryonic stem (ES), trophectoderm stem (TS) and extra-embryonic endoderm (XEN) cells lines were pulse-labelled with BrdU, stained with PI and separated by cell sorting into six cell cycle fractions according to DNA content. The gates used to define G1, S1, S2, S3, S4 and G2/M fractions of the cell cycle are indicated for each cell type. (B) Histograms showing the relative abundance of polymerase chain reaction products for early and late replicating controls (α-*globin *and *Amylase 2.1*, respectively) in each cell cycle fraction for ES (black bars), TS (white bars) and XEN (grey bars) cell lines. *Drosophila melanogaster Gbe *is an internal control for uniform recovery of BrdU-labelled DNA. Mean and standard deviation of two or more experiments are shown for each cell type analysed. Click here for file

Additional file 3 **Expression analysis of lineage-specific genes in embryonic stem (ES), trophectoderm stem (TS) and extra-embryonic endoderm (XEN) cells**. Total RNA was isolated from ES, TS and XEN cells lines, reverse-transcribed and analysed by reverse transcription polymerase chain reaction using primers for several lineage-specific markers. *Gapdh *was used as a loading control. +/-, with or without reverse transcriptase. Control tissues used as positive controls: embryoid bodies differentiated for 2.5 days (*Fgf5*), embryonic heads E12.5 (*Mash1*, *Math1*, *Neurod *and *Sox1*) and embryonic placenta E12.5 (*Pl1 *and *Tpbpa*). Click here for file

Additional File 4 **Quantification of lineage-restricted transcription factors in embryo-derived stem cells**. The relative expression of *Oct4, Nanog, Cripto, Rex1, Sox2, Cdx2, Eomes, Hand1, Gata4, Gata6, FoxA2 *and *Hnf4 *was assessed by quantitative reverse transcription polymerase chain reaction in embryonic stem (black bars), trophectoderm stem (white bars) and extra-embryonic endoderm (grey) cells. Developmental regulators characteristic of each cell type are highlighted with a blue box. Data was normalised to *Gapdh *expression. Values shown are the mean from three independent experiments and error bars indicate standard deviations. Click here for file

## References

[B1] Johnson MH Ziomek CA The foundation of two distinct cell lineages within the mouse morula Cell 1981 24 71 80 10.1016/0092-8674(81)90502-X 7237545

[B2] Chazaud C amanaka Y Pawson T Rossant J Early lineage segregation between epiblast and primitive endoderm in mouse blastocysts through the Grb2-MAPK pathway Dev Cell 2006 10 615 624 10.1016/j.devcel.2006.02.020 16678776

[B3] Rossant J Tam PP Blastocyst lineage formation, early embryonic asymmetries and axis patterning in the mouse Development (Cambridge, England) 2009 136 701 713 19201946 10.1242/dev.017178

[B4] Nichols J Zevnik B Anastassiadis K Niwa H Klewe-Nebenius D Chambers I Scholer H Smith A Formation of pluripotent stem cells in the mammalian embryo depends on the POU transcription factor Oct4 Cell 1998 95 379 391 10.1016/S0092-8674(00)81769-9 9814708

[B5] Strumpf D Mao CA Yamanaka Y Ralston A Chawengsaksophak K Beck F Rossant J Cdx2 is required for correct cell fate specification and differentiation of trophectoderm in the mouse blastocyst Development 2005 132 2093 2102 10.1242/dev.01801 15788452

[B6] Morrisey EE Tang Z Sigrist K Lu MM Jiang F Ip HS Parmacek MS GATA6 regulates HNF4 and is required for differentiation of visceral endoderm in the mouse embryo Genes Dev 1998 12 3579 3590 10.1101/gad.12.22.3579 9832509 PMC317242

[B7] Plusa B Frankenberg S Chalmers A Hadjantonakis AK Moore CA Papalopulu N Papaioannou VE Glover DM Zernicka-Goetz M Downregulation of Par3 and aPKC function directs cells towards the ICM in the preimplantation mouse embryo J Cell Sci 2005 118 505 515 10.1242/jcs.01666 15657073

[B8] Larue L Ohsugi M Hirchenhain J Kemler R E-cadherin null mutant embryos fail to form a trophectoderm epithelium Proc Natl Acad Sci USA 1994 91 8263 8267 10.1073/pnas.91.17.8263 8058792 PMC44586

[B9] Kan NG Stemmler MP Junghans D Kanzler B de Vries WN Dominis M Kemler R Gene replacement reveals a specific role for E-cadherin in the formation of a functional trophectoderm Development 2007 134 31 41 10.1242/dev.02722 17138661

[B10] Torres-Padilla ME Parfitt DE Kouzarides T Zernicka-Goetz M Histone arginine methylation regulates pluripotency in the early mouse embryo Nature 2007 445 214 218 10.1038/nature05458 17215844 PMC3353120

[B11] Chapman V Forrester L Sanford J Hastie N Rossant J Cell lineage-specific undermethylation of mouse repetitive DNA Nature 1984 307 284 286 10.1038/307284a0 6694730

[B12] Rossant J Sanford JP Chapman VM Andrews GK Undermethylation of structural gene sequences in extraembryonic lineages of the mouse Dev Biol 1986 117 567 573 10.1016/0012-1606(86)90325-8 2428685

[B13] Santos F Hendrich B Reik W Dean W Dynamic reprogramming of DNA methylation in the early mouse embryo Dev Biol 2002 241 172 182 10.1006/dbio.2001.0501 11784103

[B14] Santos F Zakhartchenko V Stojkovic M Peters A Jenuwein T Wolf E Reik W Dean W Epigenetic marking correlates with developmental potential in cloned bovine preimplantation embryos Curr Biol 2003 13 1116 1121 10.1016/S0960-9822(03)00419-6 12842010

[B15] Okamoto I Otte AP Allis CD Reinberg D Heard E Epigenetic dynamics of imprinted X inactivation during early mouse development Science 2004 303 644 649 10.1126/science.1092727 14671313

[B16] Mak W Nesterova TB de Napoles M Appanah R Yamanaka S Otte AP Brockdorff N Reactivation of the paternal X chromosome in early mouse embryos Science 2004 303 666 669 10.1126/science.1092674 14752160

[B17] Ng RK Dean W Dawson C Lucifero D Madeja Z Reik W Hemberger M Epigenetic restriction of embryonic cell lineage fate by methylation of Elf5 Nat Cell Biol 2008 10 1280 1290 10.1038/ncb1786 18836439 PMC2635539

[B18] Farthing CR Ficz G Ng RK Chan CF Andrews S Dean W Hemberger M Reik W Global mapping of DNA methylation in mouse promoters reveals epigenetic reprogramming of pluripotency genes PLoS Genet 2008 4 e1000116 10.1371/journal.pgen.1000116 18584034 PMC2432031

[B19] Azuara V Perry P Sauer S Spivakov M Jorgensen HF John RM Gouti M Casanova M Warnes G Merkenschlager M Fisher AG Chromatin signatures of pluripotent cell lines Nat Cell Biol 2006 8 532 538 10.1038/ncb1403 16570078

[B20] Bernstein BE Mikkelsen TS Xie X Kamal M Huebert DJ Cuff J Fry B Meissner A Wernig M Plath K Jaenisch R Wagschal A Feil R Schreiber SL Lander ES A bivalent chromatin structure marks key developmental genes in embryonic stem cells Cell 2006 125 315 326 10.1016/j.cell.2006.02.041 16630819

[B21] Tanaka S Kunath T Hadjantonakis AK Nagy A Rossant J Promotion of trophoblast stem cell proliferation by FGF4 Science 1998 282 2072 2075 10.1126/science.282.5396.2072 9851926

[B22] Kunath T Arnaud D Uy GD Okamoto I Chureau C Yamanaka Y Heard E Gardner RL Avner P Rossant J Imprinted X-inactivation in extra-embryonic endoderm cell lines from mouse blastocysts Development 2005 132 1649 1661 10.1242/dev.01715 15753215

[B23] Billon N Jolicoeur C Ying QL Smith A Raff M Normal timing of oligodendrocyte development from genetically engineered, lineage-selectable mouse ES cells J Cell Sci 2002 115 3657 3665 10.1242/jcs.00049 12186951

[B24] Tanaka TS Kunath T Kimber WL Jaradat SA Stagg CA Usuda M Yokota T Niwa H Rossant J Ko MS Gene expression profiling of embryo-derived stem cells reveals candidate genes associated with pluripotency and lineage specificity Genome Res 2002 12 1921 1928 10.1101/gr.670002 12466296 PMC187571

[B25] Zhang X Zhang J Wang T Esteban MA Pei D Esrrb activates Oct4 transcription and sustains self-renewal and pluripotency in embryonic stem cells J Biol Chem 2008 283 35825 35833 10.1074/jbc.M803481200 18957414

[B26] Hansen RS Canfield TK Lamb MM Gartler SM Laird CD Association of fragile X syndrome with delayed replication of the FMR1 gene Cell 1993 73 1403 1409 10.1016/0092-8674(93)90365-W 8324827

[B27] Azuara V Brown KE Williams RR Webb N Dillon N Festenstein R Buckle V Merkenschlager M Fisher AG Heritable gene silencing in lymphocytes delays chromatid resolution without affecting the timing of DNA replication Nat Cell Biol 2003 5 668 674 10.1038/ncb1006 12833066

[B28] Gilbert DM Replication timing and transcriptional control: beyond cause and effect Curr Opin Cell Biol 2002 14 377 383 10.1016/S0955-0674(02)00326-5 12067662

[B29] Simon I Tenzen T Mostoslavsky R Fibach E Lande L Milot E Gribnau J Grosveld F Fraser P Cedar H Developmental regulation of DNA replication timing at the human beta globin locus Embo J 2001 20 6150 6157 10.1093/emboj/20.21.6150 11689454 PMC125288

[B30] Cimbora DM Schubeler D Reik A Hamilton J Francastel C Epner EM Groudine M Long-distance control of origin choice and replication timing in the human beta-globin locus are independent of the locus control region Mol Cell Biol 2000 20 5581 5591 10.1128/MCB.20.15.5581-5591.2000 10891496 PMC86017

[B31] Cobb BS Nesterova TB Thompson E Hertweck A O'Connor E Godwin J Wilson CB Brockdorff N Fisher AG Smale ST Merkenschlager M T cell lineage choice and differentiation in the absence of the RNase III enzyme Dicer J Exp Med 2005 201 1367 1373 10.1084/jem.20050572 15867090 PMC2213187

[B32] Hiratani I Ryba T Itoh M Yokochi T Schwaiger M Chang CW Lyou Y Townes TM Schubeler D Gilbert DM Global reorganization of replication domains during embryonic stem cell differentiation PLoS Biol 2008 6 e245 10.1371/journal.pbio.0060245 18842067 PMC2561079

[B33] Naya FJ Huang HP Qiu Y Mutoh H DeMayo FJ Leiter AB Tsai MJ Diabetes, defective pancreatic morphogenesis, and abnormal enteroendocrine differentiation in BETA2/neuroD-deficient mice Genes Dev 1997 11 2323 2334 10.1101/gad.11.18.2323 9308961 PMC316513

[B34] Bielinska M Narita N Wilson DB Distinct roles for visceral endoderm during embryonic mouse development Int J Dev Biol 1999 43 183 205 10410899

[B35] Schotta G Lachner M Sarma K Ebert A Sengupta R Reuter G Reinberg D Jenuwein T A silencing pathway to induce H3-K9 and H4-K20 trimethylation at constitutive heterochromatin Genes Dev 2004 18 1251 1262 10.1101/gad.300704 15145825 PMC420351

[B36] Ying QL Nichols J Evans EP Smith AG Changing potency by spontaneous fusion Nature 2002 416 545 548 10.1038/nature729 11932748

[B37] Tada M Takahama Y Abe K Nakatsuji N Tada T Nuclear reprogramming of somatic cells by in vitro hybridization with ES cells Curr Biol 2001 11 1553 1558 10.1016/S0960-9822(01)00459-6 11591326

[B38] Tada M Tada T Lefebvre L Barton SC Surani MA Embryonic germ cells induce epigenetic reprogramming of somatic nucleus in hybrid cells Embo J 1997 16 6510 6520 10.1093/emboj/16.21.6510 9351832 PMC1170256

[B39] Miller RA Ruddle FH Pluripotent teratocarcinoma-thymus somatic cell hybrids Cell 1976 9 45 55 10.1016/0092-8674(76)90051-9 61820

[B40] Pereira CF Terranova R Ryan NK Santos J Morris KJ Cui W Merkenschlager M Fisher AG Heterokaryon-based reprogramming of human B lymphocytes for pluripotency requires Oct4 but not Sox2 PLoS Genet 2008 4 e1000170 10.1371/journal.pgen.1000170 18773085 PMC2527997

[B41] Stock JK Giadrossi S Casanova M Brookes E Vidal M Koseki H Brockdorff N Fisher AG Pombo A Ring1-mediated ubiquitination of H2A restrains poised RNA polymerase II at bivalent genes in mouse ES cells Nat Cell Biol 2007 9 1428 1435 10.1038/ncb1663 18037880

[B42] Guenther MG Levine SS Boyer LA Jaenisch R Young RA A chromatin landmark and transcription initiation at most promoters in human cells Cell 2007 130 77 88 10.1016/j.cell.2007.05.042 17632057 PMC3200295

[B43] Lee TI Jenner RG Boyer LA Guenther MG Levine SS Kumar RM Chevalier B Johnstone SE Cole MF Isono K Koseki H Fuchikami T Abe K Murray HL Zucker JP Yuan B Bell GW Herbolsheimer E Hannett NM Sun K Odom DT Otte AP Volkert TL Bartel DP Melton DA Gifford DK Jaenisch R Young RA Control of developmental regulators by Polycomb in human embryonic stem cells Cell 2006 125 301 313 10.1016/j.cell.2006.02.043 16630818 PMC3773330

[B44] Boyer LA Plath K Zeitlinger J Brambrink T Medeiros LA Lee TI Levine SS Wernig M Tajonar A Ray MK Bell GW Otte AP Vidal M Gifford DK Young RA Jaenisch R Polycomb complexes repress developmental regulators in murine embryonic stem cells Nature 2006 441 349 353 10.1038/nature04733 16625203

[B45] Spivakov M Fisher AG Epigenetic signatures of stem-cell identity Nat Rev Genet 2007 8 263 271 10.1038/nrg2046 17363975

[B46] Endoh M Endo TA Endoh T Fujimura Y Ohara O Toyoda T Otte AP Okano M Brockdorff N Vidal M Koseki H Polycomb group proteins Ring1A/B are functionally linked to the core transcriptional regulatory circuitry to maintain ES cell identity Development 2008 135 1513 1524 10.1242/dev.014340 18339675

[B47] Lehtonen E Laasonen A Tienari J Teratocarcinoma stem cells as a model for differentiation in the mouse embryo Int J Dev Biol 1989 33 105 115 2485690

[B48] Yang Q Bermingham NA Finegold MJ Zoghbi HY Requirement of Math1 for secretory cell lineage commitment in the mouse intestine Science 2001 294 2155 2158 10.1126/science.1065718 11739954

[B49] Hogan BL Barlow DP Kurkinen M Reichert's membrane as a model for studying the biosynthesis and assembly of basement membrane components Ciba Found Symp 1984 108 60 74 6569831 10.1002/9780470720899.ch5

[B50] Cross JC Baczyk D Dobric N Hemberger M Hughes M Simmons DG Yamamoto H Kingdom JC Genes, development and evolution of the placenta Placenta 2003 24 123 130 10.1053/plac.2002.0887 12596737

[B51] Hattori N Nishino K Ko YG Ohgane J Tanaka S Shiota K Epigenetic control of mouse Oct-4 gene expression in embryonic stem cells and trophoblast stem cells J Biol Chem 2004 279 17063 17069 10.1074/jbc.M309002200 14761969

[B52] Hattori N Imao Y Nishino K Ohgane J Yagi S Tanaka S Shiota K Epigenetic regulation of Nanog gene in embryonic stem and trophoblast stem cells Genes Cells 2007 12 387 396 10.1111/j.1365-2443.2007.01058.x 17352742

[B53] Johnson MH McConnell JM Lineage allocation and cell polarity during mouse embryogenesis Semin Cell Dev Biol 2004 15 583 597 10.1016/j.semcdb.2004.04.002 15271304

[B54] Odom DT Dowell RD Jacobsen ES Gordon W Danford TW MacIsaac KD Rolfe PA Conboy CM Gifford DK Fraenkel E Tissue-specific transcriptional regulation has diverged significantly between human and mouse Nat Genet 2007 39 730 732 10.1038/ng2047 17529977 PMC3797512

[B55] Adewumi O Aflatoonian B Ahrlund-Richter L Amit M Andrews PW Beighton G Bello PA Benvenisty N Berry LS Bevan S Blum B Brooking J Chen KG Choo AB Churchill GA Corbel M Damjanov I Draper JS Dvorak P Emanuelsson K Fleck RA Ford A Gertow K Gertsenstein M Gokhale PJ Hamilton RS Hampl A Healy LE Hovatta O Characterization of human embryonic stem cell lines by the International Stem Cell Initiative Nat Biotechnol 2007 25 803 816 10.1038/nbt1318 17572666

