# Supplementary figures and images for: Differences in the epigenetic and reprogramming properties of pluripotent and extra-embryonic stem cells implicate chromatin remodelling as an important early event in the developing mouse embryo
Source: Epigenetics Chromatin. 2010 Jan 12;3:1. doi: 10.1186/1756-8935-3-1 (PMC2821315; doi:10.1186/1756-8935-3-1)

**A**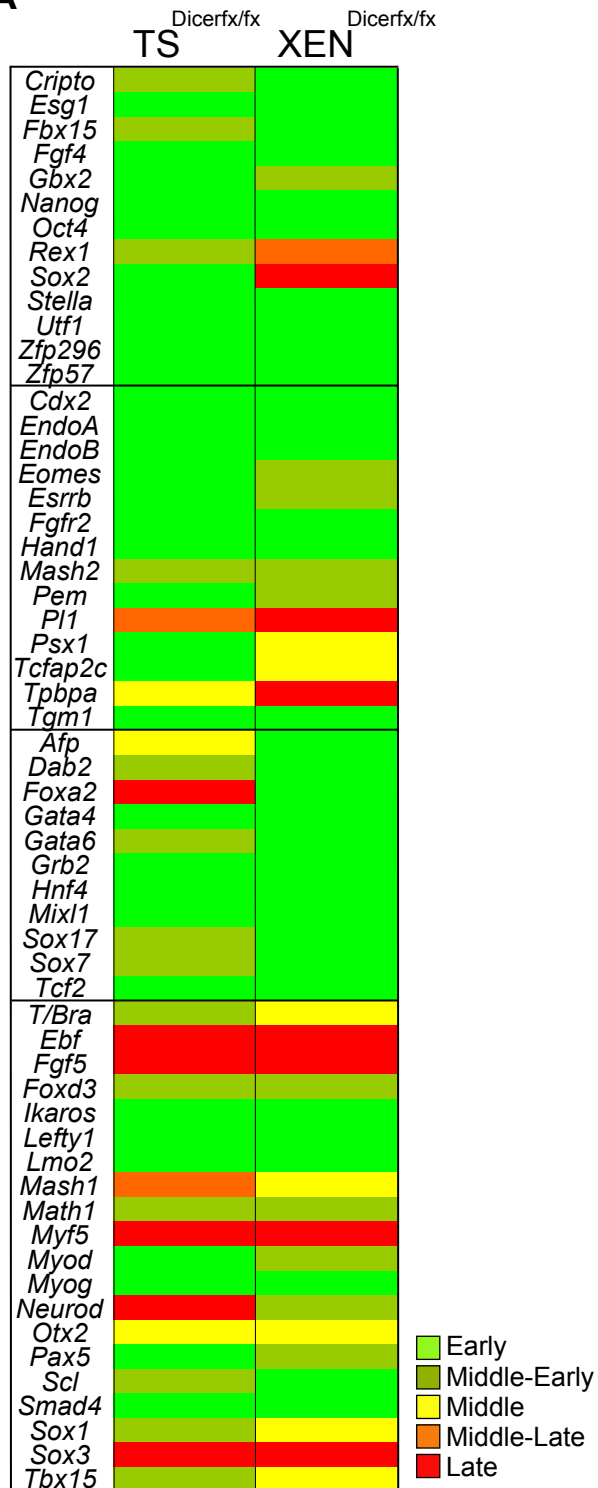**B**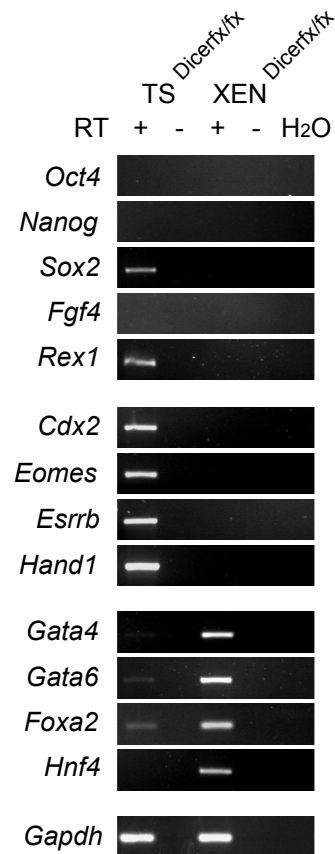

Supplement: Additional file 1 — Replication timing and gene expression analysis of additional trophectoderm stem (TS) and extra-embryonic endoderm (XEN) cell lines. (A) Summary of the replication timing comparison of the selected candidate genes in TSDicerfx/fx and XENDicerfx/fx stem cell lines. The replication timing of each gene was defined according to its peak abundance in G1/S1 (early, dark green), S2 (middle-early, light green), S2 and S3 (middle, yellow), S3 (middle-late, orange) or S4/G2 (late, red), determined in at least two independent experiments. Inner cell mass/ES-, trophectoderm/TS-, primitive endoderm/XEN-related loci or genes involved in the specification of somatic cell types are grouped into four different boxes. Comparison with data presented in Figure 2A shows that 50/58 and 52/58 showed identical replication times in TS and XEN cells, respectively. Of the remaining genes, 8/8 and 6/6 showed similar replication times (peak abundance in an adjacent cell cycle fraction) in these cell lines. (B) Reverse transcriptase polymerase chain reaction expression analysis of TSDicerfx/fx and XENDicerfx/fx cell lines. RNA was isolated and cDNA analysed using primers for Oct4, Nanog, Sox2, Fgf4, Rex1, Cdx2, Eomes, Esrrb, Hand1, Gata4, Gata6, Foxa2, Hnf4 and Gapdh as a loading control. +/- indicates presence or absence of reverse transcriptase; H2O, water control. [file 1756-8935-3-1-S1.PDF]

**A**

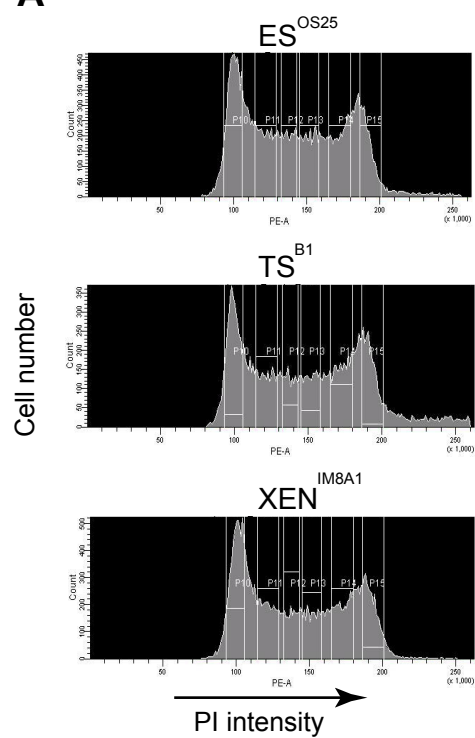

**B**

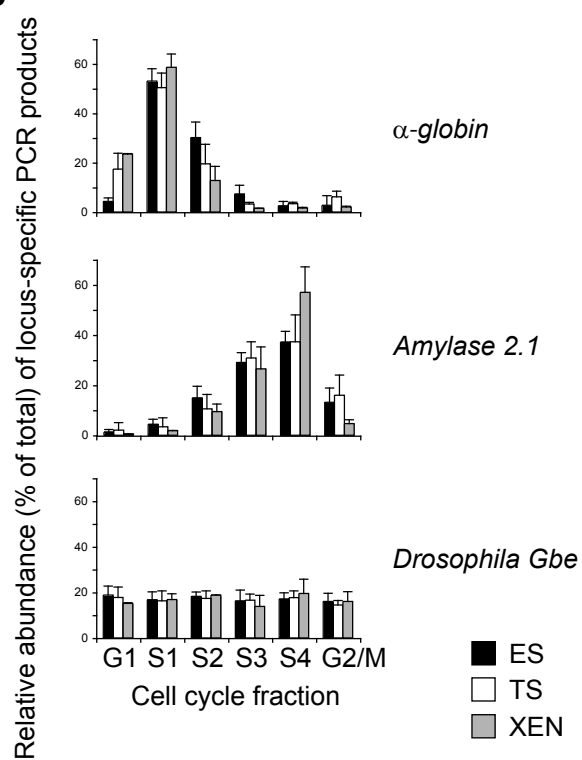

Supplement: Additional file 2 — Replication timing analysis of embryo-derived stem cell lines. (A) Typical cell cycle profiles for each cell population based on propidium iodide (PI) staining. Embryonic stem (ES), trophectoderm stem (TS) and extra-embryonic endoderm (XEN) cells lines were pulse-labelled with BrdU, stained with PI and separated by cell sorting into six cell cycle fractions according to DNA content. The gates used to define G1, S1, S2, S3, S4 and G2/M fractions of the cell cycle are indicated for each cell type. (B) Histograms showing the relative abundance of polymerase chain reaction products for early and late replicating controls (α-globin and Amylase 2.1, respectively) in each cell cycle fraction for ES (black bars), TS (white bars) and XEN (grey bars) cell lines. Drosophila melanogaster Gbe is an internal control for uniform recovery of BrdU-labelled DNA. Mean and standard deviation of two or more experiments are shown for each cell type analysed. [file 1756-8935-3-1-S2.PDF]

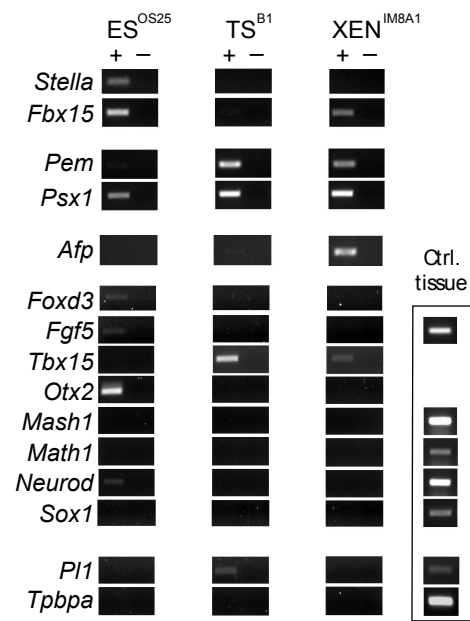

Supplement: Additional file 3 — Expression analysis of lineage-specific genes in embryonic stem (ES), trophectoderm stem (TS) and extra-embryonic endoderm (XEN) cells. Total RNA was isolated from ES, TS and XEN cells lines, reverse-transcribed and analysed by reverse transcription polymerase chain reaction using primers for several lineage-specific markers. Gapdh was used as a loading control. +/-, with or without reverse transcriptase. Control tissues used as positive controls: embryoid bodies differentiated for 2.5 days (Fgf5), embryonic heads E12.5 (Mash1, Math1, Neurod and Sox1) and embryonic placenta E12.5 (Pl1 and Tpbpa). [file 1756-8935-3-1-S3.PDF]

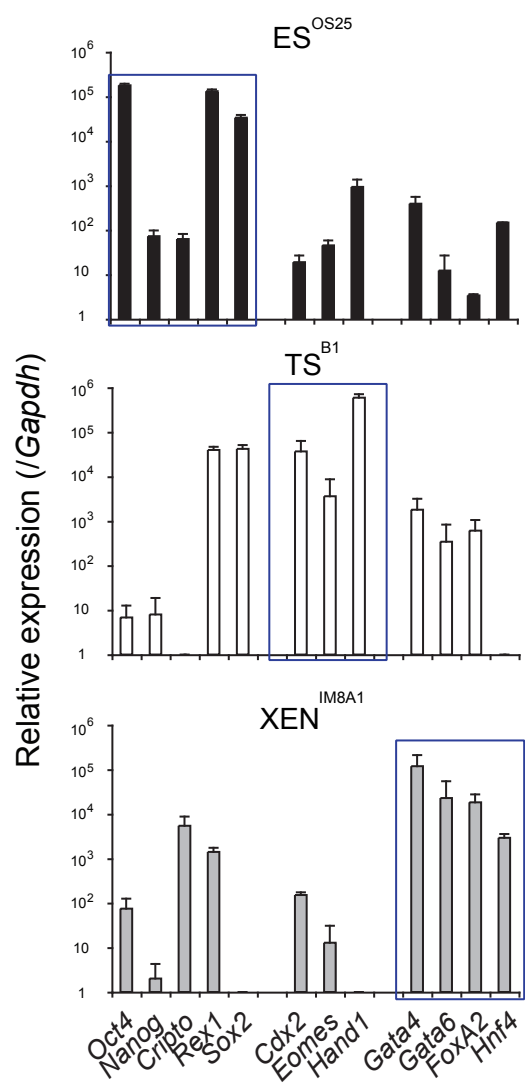

Supplement: Additional File 4 — Quantification of lineage-restricted transcription factors in embryo-derived stem cells. The relative expression of Oct4, Nanog, Cripto, Rex1, Sox2, Cdx2, Eomes, Hand1, Gata4, Gata6, FoxA2 and Hnf4 was assessed by quantitative reverse transcription polymerase chain reaction in embryonic stem (black bars), trophectoderm stem (white bars) and extra-embryonic endoderm (grey) cells. Developmental regulators characteristic of each cell type are highlighted with a blue box. Data was normalised to Gapdh expression. Values shown are the mean from three independent experiments and error bars indicate standard deviations. [file 1756-8935-3-1-S4.PDF]
